# Improvement of Presbyopia Using a Mixture of Traditional Chinese Herbal Medicines, Including Cassiae Semen, Wolfberry, and *Dendrobium huoshanense*

**DOI:** 10.1155/2021/9902211

**Published:** 2021-07-27

**Authors:** Chi-Ting Horng, Jui-Wen Ma, Po-Chuen Shieh

**Affiliations:** ^1^Department of Ophthalmology, Fooying University Hospital, Pintung 928, Taiwan; ^2^Department of Pharmacy, Tajen University, Pingtung 907, Taiwan; ^3^Unique Biotechnology Co., Ltd., Koahsiung 800, Taiwan

## Abstract

**Background:**

Presbyopia is a primary cause of a decline in near vision. In this study, we developed a new mixed herbal medicine to retard presbyopic progression and increase the amplitude of accommodation (AA), which is beneficial for near vision.

**Methods:**

A total of 400 participants between the ages of 45 and 70 years were recruited. We designed the mixed herbal drug to include Cassiae Semen (200 mg), wolfberry (200 mg), and *Dendrobium huoshanense* (DD) (40 mg) in one capsule. In experiment 1, the recruited subjects were directed to perform a push-up test to measure their AA; this was then converted to the additional diopters of reading glasses. In experiment 2, 240 subjects took three capsules daily for six months and then stopped medical therapy for a six-month follow-up. In experiment 3, 160 subjects were randomly categorized into four groups: a placebo group, low-dose group (LDG) (1 capsule daily), middle-dose group (MDG) (two capsules daily), and high-dose group (HDG) (three capsules daily). The 160 volunteers took different doses for six months and then stopped treatment, accompanied by another six-month follow-up. In experiments 2 and 3, the change in AA, uncorrected far visual acuity (UFVA), and uncorrected near visual acuity (UNVA) were recorded each month for one year.

**Results:**

In experiment 1, AA was found to decrease with age and a great deal of additional power was needed in older individuals. In experiment 2, the mean AA reached a maximum value of 2.1D (*P* < 0.05) after six months, while the UNVA improved by about two to three lines of a Jaeger chart in most of the subjects. At nine months, all the means decreased slightly to 2.0 D (*P* < 0.05). This meant that the mixed herbal medicine could still maintain AA for another three months because the herbal therapy was stopped at the seventh month. In experiment 3, the maximal AA was 2.8D, 2.9D, and 3.2D (*P* < 0.05) in the LDG, MDG, and HDG after six-month treatments, respectively. Experiment 3 showed that AA gain occurred in a dose-dependent manner; the higher the dose, the greater the AA value.

**Conclusion:**

Only two studies on the use of herbal drugs for presbyopia have been reported in PubMed. In our study, we found that taking a mixed herbal drug caused an excellent gain in AA. This is the first study to report that the characteristics of the new herbal regimen could retard and even ameliorate presbyopia.

## 1. Background

The prevalence of presbyopia in the population is gradually growing worldwide. Its prevalence was estimated to be at 1.7–2.0 billion people globally in 2015. A total of 826 million of those affected do not have sufficient vision correction. In China, ≥12% of patients with vision impairment are a result of uncorrected presbyopia [1]. Presbyopia typically affects people from the 4th decade of life; however, accommodation to it decreases with age, with it nearly finishing after age fifty and impacting almost 100% of people over the age of 65. It is worth noting that, in recent years, the overuse of smartphones and portable computers has led to the rapid progression and onset of presbyopia (around 35 years). The mechanisms of accommodation depend on the contraction of the ciliary muscle and iris; changes in the shape of the lens and convergence can show a variation in dioptric powers to enable people to see or work easily at near distances [2].

When people gaze at objects intently, the accommodation initiates a series of actions. Accommodation is the ability of the eye to change focus, and the amplitude of accommodation (AA) is the maximum potential increase in diopter that an eye can achieve in adjusting its focus. The accommodative system is controlled by acetylcholine (Ach) from the parasympathetic nerves. The change in biometry that diopters exert and increasing lenticular stiffness are responsible for presbyopia. AA should be measured corresponding to the acuity at the viewing distance. When a human wants to see objects about 40 cm in front of the eyes and the accommodation is functional, the ciliary muscles began to contract, anchor at the scleral spur, and the trabecular meshwork with peripheral cornea comes forward. Lastly, the thickness of the lens increases, and the person is able to view nearby objects. Presbyopia is a physiologic inevitability and aging is the primary etiology. With the development of society, the overuse of portable computers and smartphones has become an important cause of presbyopia [3, 4]. Geographic and sex factors have also been reported [5, 6]. In general, presbyopia manifests at 40–45 years of age due to the gradual loss of accommodation, necessitating the extra-additional (Add) power of reading spectacles. AA generally decreases at a rate of 0.3 D per year. The symptoms of presbyopia usually occur when the AA value decreases to below about 3–4 D. At this point, subjects with presbyopia suffer from blurred vision, asthenopia, ocular pain, and headaches after reading or working at close distances for prolonged periods. In our current understanding of the principles, the add diopter is about 0.097 to 0.105 D/year for presbyopia. When patients are 45 to 50 years of age, the decline in accommodation is around 3 D/year. By the fifth decade of life, there is an obvious reduction in AA. It is not surprising that this accommodation decreases to nearly zero at the age of 70 years [7].

Except for AA, presbyopia occurs due to changes in the chemical composition and physical structure of the lens [8]. There exists a close relationship between the biomechanics of the lens, accommodative system, and presbyopia. The thickness of the elastic capsule of the lens and the flexibility of zonular fibers change during adulthood may impact patients with presbyopia. An increase in the fibrillary materials of zonules reduces the compliance of the posterior insertion of the ciliary muscle. The deterioration of the elastic components of the ciliary body and choroid may result in a decline in AA. Furthermore, vitreous liquefaction occurs and the pressure between the anterior and posterior chamber may change with age [9]. The irregularity of the fibrous morphology of the lens is also noted when humans become older. These findings have been reported as the predisposing factors for presbyopia [10]. Hence, the loss of AA in individuals begins early in life at 40 to 50 years of age and rapidly progresses when people are around 55 years old.

Presbyopia is also believed to reflect a loss of accommodation due to cataract formation and the weakening of the ciliary muscles—the lens zonules apparatus. According to Hemohotz (1855), the ciliary muscles relax and the zonules around the lens are under initial tension. When the eye accommodates, the tractive force from the ciliary muscles enhances and reduces the tension on the zonules. The reduced zonular tension allows the elastic capsule of the lens to contract, causing an increase in the anterior–posterior diameter of the lens, making it more spherical in shape. Finally, it may increase the diopter powers needed for viewing near objects. Therefore, Schachar et al. demonstrated that, during accommodation, the central surface should steepen while the peripheral surface of the crystalline lens flattens [8]. Nandi et al. also proposed two major probable factors related to presbyopia—the contractility of the ciliary muscles and the resistance to deformation of the compacted lenticular sclerosis due to the deposition of damaged fibers and the cross-linking of the *α*-crystalline protein through the formation of advanced glycation end-products (AGEs) [11]. Hence, the diopter of the lens may reduce gradually because of the excessive accumulation of AGEs for oxidation, which leads to a harder nucleus. On the other hand, Pescosolido and colleagues demonstrated that the ciliary muscles undergo compensatory hypertrophy and AA decreases with age because of the loss of the elasticity and ductility of muscles [12]. There are several procedures available for evaluating the outcomes of treatments of presbyopia. The evaluation parameters include the change in refraction, pupil size, axial length, near vision, depth of the anterior chamber (A/C), lens thickness, and level of AA. Schneider et al. made use of the Hartinger coincidence refractometer to examine the A/C depth and pupillary diameter between accommodative intraocular lenses (IOL) and PMMA IOL of 30 eyes after cataract surgery. The aim of their research was to identify many factors in different IOLs and make a contribution to the development of IOL in humans [13]. Additionally, the change in AA was valuable in assessing the prognosis. For instance, a decrease in the AA of the nondominant eye should be the predictive factor for the success rate of exotropia surgery [14].

When accommodation is not enough for people at near working distances, some auxiliary procedures are necessary for avoiding vision stress and ocular disability. Common strategies for coping with presbyopia are the use of reading glasses and contact lenses. Moreover, other procedures have also been claimed to restore accommodative ability, including orthokeratology, corneal inlays, Supracor laser procedure, cataract surgery with multifocal IOLs, PresbyLASIK, diode laser thermal keratoplasty, surgical expansion of the sclera, and conductive keratopathy [15]. Although spectacles can meet the basic needs of the majority of individuals, they may be inconvenient to use. In addition, some of these surgeries are either undeveloped or controversial and several of the methods are not acceptable because of their invasive and relatively dangerous properties. Therefore, there is a need to develop a relatively safe, new, and effective method to address presbyopia.

Pharmacologic therapy for presbyopia is another choice, which shows the characteristics of reversibility, noninvasiveness, and ease of use. From 2005 to 2016, very few articles about pharmacologic treatment for presbyopia were published. For example, Abdelkader stated that a combination of eye drops with 3% carbachol (parasympathomimetic agent) and 0.2% brimonidine (*α*2-adrenergic agonist) was prescribed for 10 subjects with presbyopia between the ages of 42 and 58 years, once daily. Carbachol should promote Ach release and lead to miosis to enhance depth-of-focus (DoF) and the pinhole effect, thus obtaining accommodative capacity in subjects between their forties and fifties. Brimonidine enhances the effects of carbachol and produces a more miotic pupil. A statistically significant improvement in mean near vision (4-lines) was achieved in all persons, while far vision was still preserved [16]. Renna et al. claimed that their ophthalmic formulation with 0.247% pilocarpine (cholinergic agonist), 0.78% phenylephrine (*α*1-adrenergic agonist), 0.023% nepafenac (NSAID), and 0.003% naphazoline (sympathomimetic agent) can be recommended to patients with presbyopia (28 eyes) twice-a-day binocularly. The mechanisms of the complex formulation are ciliary muscle contraction, lens softening, and miosis. Pilocarpine predominantly stimulates accommodation and enables miosis and ciliary body contraction. It also improves the tear volume by enhancing lacrimal gland secretion, which relieves the effects of ocular fatigue and dry eye. The outcome showed a mean improvement in UNVA of two to three lines by J chart [17].

In recent years, the worldwide use of herbal medicines for eye diseases has become popular and trendy. For example, *Astragalus membranaceus* can be used for diabetic retinopathy, while *Cinnamon cassia* could be beneficial for age-related macular degeneration [18]. We reviewed the topic of herbal treatment for presbyopia from studies reported in PubMed, wherein only two studies were found. Our team has published reports examining the use of cassia seeds and *Lycium barbarum* for eye diseases [19, 20]. In this new study, our mixed formulation of herbal drugs to address presbyopia included Cassiae Semen, *L. barbarum*, and *Dendrobium huoshanense* (DD). We collected the extracted substances, ground them into a fine powder, and mixed them in a capsule according to a specific ratio [21]. In the current study, we attempted to use these three herbal drugs on a large sample of subjects and investigated the therapeutic effects.

## 2. Methods

In this approved study, we selected 400 consecutive eyes (200 right eyes and 200 left eyes) randomly from 400 adult participants aged 45 to 70 who could not easily perform near work (around 40 cm) in southern Taiwan. We conducted this prospective study in 2016, after obtaining informed consent from all subjects. Then, investigations were conducted in accordance with the Declaration of Helsinki. The ethical approval for the human trial was obtained from the institutional review board of Kaohsiung Armed Forces General Hospital (Kaohsiung City, Taiwan, ROC) (approval number: KAFGH-106-003). All patients with presbyopia underwent a standardized protocol of ocular examination, including slit-lamp biomicroscopy (Haag-Streit IM 900, Clinico. INC), noncontact tonometry (Reichert® 7, Reichert Technol), autorefraction (KA-1000, KOWA), and nonmydriatic retinal photography (Nonmyd AF, KOWA) for checking the cornea, lens, A/C depth, intraocular pressure (IOP), and refraction. An optical coherence tomography system (Zeiss Com.) was applied to evaluate the vitreous, retina, and optic nerve. The horizontal diameter of pupils was measured using Orbscan II Topography (Bausch & Lomb). Only emmetropia (cycloplegic spherical equivalent, ±1.0D; astigmatism ≤0.5D) measured from an autorefractor was used. For example, longer axial myopia has a thicker ciliary body, which impacts the accommodation of presbyopia and adversely affects the results. Hence, only subjects with emmetropic eyes were enrolled to avoid bias [22]. The uncorrected distance visual acuity (UDVA) and uncorrected near visual acuity (UNVA) were examined. Particular attention was given to the UDVA according to the Snellen chart (test distance: 6 mm). UNVA was assessed by the Jaeger Eye Chart (test distance: 40 cm; J score: 1–15), where people with presbyopia were told to read the letters of books until the letters are no longer clearly seen without reading glasses [23]. In our experiments, all the eligible subjects with at least 20/32 UDVA and J6 UNVA were enrolled, according to another study [24]. For easy analyses, UDVA was transferred to LogMAR. Volunteers with ocular pathologies such as glaucoma, history of eye trauma, uveitis, amblyopia, strabismus, severe cataracts (Grade III and IV), severe corneal diseases, optic neuropathy, various retinopathy, s/p any major ocular surgery, receiving correction of presbyopia, and stereopsis less than 400 arc second were excluded. In addition, none of the participants had received any chronic miotic or mydriatic therapy, which could have caused an error in our results.

Several examinations were conducted for all 400 eyes. At each visit monthly, the ocular structure, IOP, and pupil diameter of all the involved eyes were checked. Additionally, we verified the change in AA to evaluate the effectiveness of the herbal treatment. There are many methods for obtaining AA in various conditions [25, 26]. The Donder table and Hofstetter's equation can be used to measure AA easily, although the accuracy of these methods remains controversial due to the omission of some facts [27]. Hence, we adopted the push-up method for assessing the AA of each subject AA using an autorefractometer, which is a more suitable manner for surveying AA. The push-up test is a valuable technique for an estimate of near reading ability because it has the advantages of the rapid and simple measurement of AA [28, 29]. Although the push-up test is a subjective method, its repeatability and validity are highly appreciated [30]. Hence, we used this equipment for a monocular AA check-up. First, the AA from each subject with different ages and conditions was checked at the baseline, in the third, sixth, ninth, and twelfth months (the end of this study). In the initial experiment, each participant sat in a room where the lights were switched off to create a semidark condition. All the measurements were taken twice, and the arithmetic mean of the results was registered. As for the detailed procedures, the refractive correction was prescribed for a far distance if necessary. Subjects had an eye patch placed over their nontested eyes and were asked to focus on a near target consisting of two parallel vertical lines (designed with a constant angular size) on a standard chart. The chart was brought closer until the target became slightly blurred. This chart was then slowly pushed back until the parallel vertical lines could be clearly seen again, which was considered to be the near point of accommodation. The reciprocal of the closest distance in meters was AA [31]. Throughout the study, all adverse effects were recorded. A questionnaire recording visual satisfaction compliance with taking drugs was administered to patients each month. The definition of success was an improvement in UNVA of at least two lines of the J chart, accompanied by presbyopic symptoms, including ocular fatigue, dry eye, and periocular pain when working at near distances after six months of treatment. The rates of satisfaction of the subjects were collected between the first and twelfth months.

According to our small sample of previous human studies, the ideal formula was a mixture of Cassiae Semen (200 mg), wolfberry (200 mg), and DD (40 mg) in a capsule. The ratio of cassia seeds, goji, and DD was 5 : 5 : 1 (not published yet). The medical plant powders were purchased from herbal drug stores in Taiwan. We manufactured a slow-releasing capsule that included the three herbal rugs together. The special components of this capsule included hydroxylpropyl methylcellulose (HPMC) and pectin. HPMC is a semisynthetic, inert, viscoelastic polymer that is used in eye drops and as an excipient for controlled-delivery compositions in oral medications. Pectin can act as a gelling agent, thickening agent, and stabilizer. In capsules, pectin should resist gastric acid and ensure that the herbal drugs are absorbed in the small intestine for maximal effect. Regarding the choice of criteria cases in our study, people aged ≥45 years were recruited. Wold et al. demonstrated that problems manifest earliest in hyperopes and emmetropes at about 40 years of age [28]. According to our clinical experience in Taiwan, the symptoms of presbyopia get remarkably worse between 40 and 45 years of age. We also found that the average age for requesting added correction for reading spectacles is around 45 years due to the significant reduction in AA. Therefore, we chose subjects whose mean age was 45 for real and exact analysis in this research.

In experiment 1,400 participants with various mean ages (45, 50, 55, 60, 65, and 70 years) were recruited. AA was measured by the push-up test and converted to the presbyopic powers of the individual [32]. In the early stages of presbyopia, residual accommodation remains crucial for achieving the functional near vision. Then, 200 participants with a mean age of 50 years were separated into 40 subjects for experiment 2 and 160 subjects for experiment 3, which will be the subject of another study.

**Figure 1:**
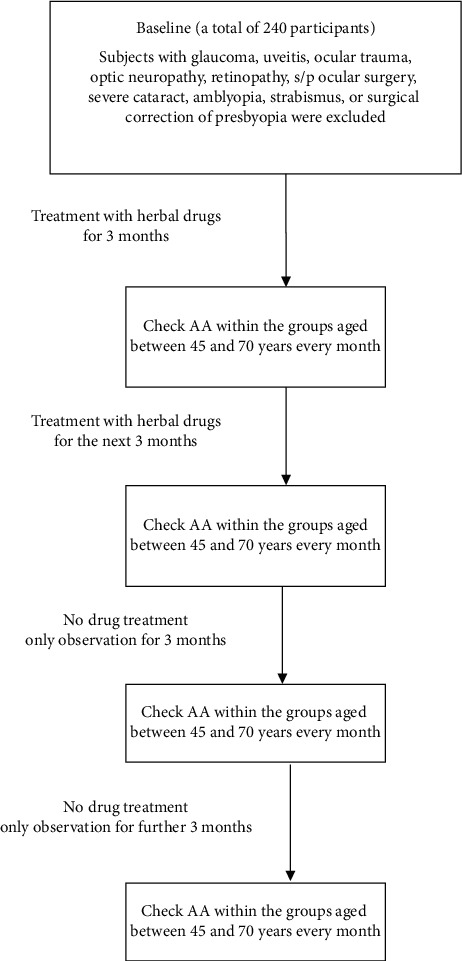
Flowchart of experiment 2.

In experiment 2, all 240 participants were categorized into six groups according to their different ages (45, 50, 55, 60, 65, and 70 years), and each group included 40 subjects. We obtained the AA from experiment 1. In the beginning, 240 patients took one capsule three times a day for six months. Afterwards, from the start of the seventh month, all the subjects stopped taking drugs and received a further six-month follow-up (Figure 1). The aim of experiment 2 was to evaluate the efficacy of these mixed herbal drugs in improving accommodation. From the start of experiment 2, the participants were requested to report to our outpatient department and participate in a push-up test to survey their AA every month. We completed the recordings, including UDVA, UNVA, IOP, and pupil diameter. We first compared the results at the baseline and at other time points (i.e., after three, six, nine, and twelve months) using Scheffe's test. We then inspected the change in all parameters at the ninth and twelfth month compared with the results at the sixth month. We also examined the difference after stopping herbal treatment using Scheffe's test. Finally, the mean IOP, horizontal diameter of the pupil, UNVA, and UNVA were analyzed among the six groups.

**Figure 2:**
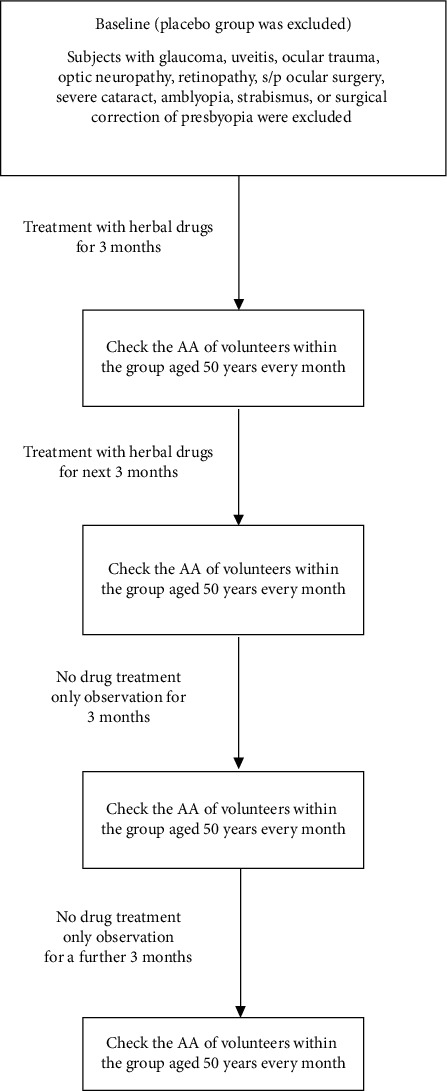
Flowchart of experiment 3.

In experiment 3, 160 presbyopic subjects with a mean age of 50 were randomly categorized into four groups, including a placebo group (taking 10 mg vitamin C daily), low-dose group (LDG) (1 capsule/day), middle-dose group (MDG) (2 capsules/day), and high-dose group (HDG) (3 capsules/day), respectively. According to our designed flow chart, the procedures used in experiments 2 and 3 were similar. Oral vitamin C was prescribed for placebo group 1. Moreover, in groups 2, 3, and 4, the volunteers took one, two, and three capsules every day, respectively, for six months (Figure 2). When half a year had passed, the presbyopic patients terminated the use of the herbal supplements and received follow-up for the next six months. We compared the parameters, including AA, pupil size, UNVA, and UDVA at the baseline and at other times (third, sixth, ninth, and twelfth months) using Scheffe's test. Then, we checked all the parameters at the ninth and twelfth months and compared them to the sixth month. We inspected the difference after the discontinuation of herbal drugs using Scheffe's test. We also measured the difference in AA and other values between the placebo and other three groups (LCG, MCG, and HCG) at various times (i.e., the baseline and the end of the third, sixth, ninth, and twelfth months) using William's test.

The AA, pupil size, and presbyopic diopters were presented as mean ± standard deviation (SD). We analyzed the data using SAS 9.0 (SAS Institute, Cary, USA). A *P* value <0.05 was considered statistically significant when compared in all the experiments.

## 3. Results

A total of 200 males and 200 females participated in our study, and their average age was 54.5 ± 2.8 years (range: 42–72 years). Their IOP remained within a normal range after the oral intake of herbal drugs for one year. In addition, the anatomy of the ocular structure of all participants except the diameters of the pupils were all found to be normal at the sixth month and the end of our study (twelfth month). We observed the lens of every volunteer, and there was no rapid progression to severe cataract formation (grade III or IV). Additionally, there were no significant adverse events or complications—for example, severe diarrhea, red-eye, skin rash, or even convulsion—mentioned by any participants. We did not find overaccommodation-induced headaches from excessive ciliary muscle contractility. In our entire study, the percentage of good compliance and satisfaction after taking herbal capsules was up to 90% (324/360). Forty volunteers in the placebo group in experiment 3 were deductible from the total number of 400 participants. The success rate was nearly 95% (342/360) in the sixth month. In other words, the symptoms and near vision in 95% of presbyopic patients would improve after herbal treatments for a total of six months.

In experiment 1, 400 participants of different ages (45, 50, 55, 60, 65, and 70 years) completed the push-up test, and their AA values were estimated. For example, the mean AA of people aged 60 years was 1.0 ± 0.1 D, and this was converted to the add powers of +2.0 ± 0.1 D by a special formula, showing that they potentially needed reading glasses [33]. Additionally, the AA of people aged 45 and 70 years was 3.6 ± 0.1 D and 0.2 ± 0.1 D, respectively. The decrease in AA with age was verified. The level of AA in presbyopic subjects aged 65 and 70 years was approximately zero (0.2 D∼0.3 D) (Table 1). In other words, people aged ≥65 years really need the add power of eyeglasses. Hence, reading spectacles were necessary for older presbyopic persons because of their total loss of accommodative ability. Physical status (i.e., the difference in arm length), visual tasks, and working distance affect the add power for individual patients. In fact, it is a meticulous technique about the procedure of getting a prescription for a pair of reading glasses. However, Taub and Shallo-Hoffmann showed that the add power required for working at near distances is provided by a combination of spectacle addition and residual accommodation [34]. In the study, the mean AA (add power) was 3.6 ± 0.2 D (+0.7 ± 0.1 D), 2.6 ± 0.1 (+1.2 ± 0.1 D), and 1.8 ± 0.2 D (+1.6 ± 0.1 D) for people aged 45, 50, and 55 years, respectively. These results of our experiments were in agreement with the earlier published notion of presbyopia beginning at 40–45 years old and lower AA apparently occurring in the mid-fifties [35].

In experiment 2, 240 subjects were categorized into six groups in accordance with their different ages. Experiment 2 aimed to determine the outcome of using the same dose for subjects of different ages. Each patient took three capsules each day for six months and received follow-up for the next six months continuously. The findings from the various groups revealed that AA could reach the maximal level at the sixth month, perhaps because the herbal drugs reached their highest accumulated concentration in the human body in that period (Table 2). We took the subjects aged 55 years as an example. When comparing the results at the baseline, the mean AA reached the maximal level (2.3 ± 1.5 D) at the sixth month (*P* < 0.05). In addition, the highest AA values among all the six groups were around the sixth month. In summary, the herbal drugs were given to volunteers aged 45 to 70 years for six months and the AA value reached a maximum value of 2.1 D in the sixth month (*P* < 0.05) and decreased slightly to 2.0 D in the ninth month (*P* < 0.05). Furthermore, from the seventh month, the AA gradually decreased because of the decayed drug reaction. However, it was interesting to find that AA still maintained significant results in people aged 65 and 70 years in the ninth month (*P* < 0.05). Surprisingly, this was when the participants had already stopped herbal supplementation for three months, thus showing that excellent pharmacologic abilities were still achieved in the ninth month. Therefore, we propose that our designed formula could perhaps maintain valid AA and maintenance effects for patients with presbyopia.

The mean pupil diameter was 4.3 ± 1.1 mm, 5.0 ± 0.9 mm, 5.6 ± 0.8 mm, 4.5 ± 0.7 mm, and 4.2 ± 0.5 mm at the beginning of the study and the third, sixth, ninth, and twelfth months, respectively. We found that the pupil would slowly dilate to the maximal size after six months and then recover slowly to nearly the initial size. The UDVA and UNVA were 0.18 LogMAR and J5.5, 0.09 LogMAR and J3.5, 0.05 LogMAR and J1.5, 0.12 Log MAR and J2.5, and 0.17Log MAR and J5.0 at the initial phase, at the 3^rd^ month, at the third, sixth, ninth, and twelfth months, respectively. Hence, it was verified that far vision was not affected in our treatment. Besides this, in most of the participants, the near vision measured with the Jaeger Eye Chart showed an improvement of around two to three lines. The mean UNVA reached J1.5, which may be beneficial for patients with presbyopia after six-month treatment. The mean UNVA was also maintained at J2.5 (ninth month), even after the herbal supplement was stopped for three months. This meant that the mixed herbal medicine maintained its effects for good near visual acuity. Meantime, mydriasis showed the largest pupil size (5.6 ± 0.8 mm) at the sixth month (*P* < 0.05), and the diameter of the pupil showed a mild decrease (4.5 ± 0.7 mm) at the ninth month (*P* < 0.05) when compared with the baseline. The parasympathetic functions after taking our mixed herbal drugs were predominant in terms of pupillary dilation. Besides this, the maintenance doses from our herbal drugs were also appreciated for at least three months. A smaller pupil enhances the DoF and improves human near vision [36]. However, mydriasis and excellent near vision were noted after our herbal drug supplementations. We propose that the mechanisms of improvement of near visual acuity may be due to accommodation rather than miosis-induced DoF and the pinhole effect in our subjects.

In experiment 3, 160 participants with a mean age of 50 years were randomly categorized into four groups for different treatments. In this experiment, we wanted to realize the appropriate doses for patients with presbyopia. During our observation, the AA of the subjects in the placebo group who took vitamin C remained unchanged. On the other hand, we found that the maximal AA was 2.8 ± 0.4 D, 2.9 ± 0.8 D, and 3.2 ± 0.5 D in the LDG, MDG, and HDG in the sixth month (Table 3). Only the elevation of AA in HDG showed a significant difference when compared with the baseline (*P* < 0.05). The increase in AA in LDG and MDG was also apparent. Furthermore, the AA (3.0 ± 0.5 D) in HDG was still elevated at the ninth month; this difference was also significant when compared with the baseline (*P* < 0.05) using Scheffe's test. To our surprise, the AA remained good in the HDG after the subjects had stopped the treatment for three months. This meant that the residual effects of the herbal treatment in the human body still maintained a therapeutic function after the subjects had stopped continuous herbal therapy for three months. In experiment 2, we had a similar outcome and associated conclusions. We compared the results between the placebo group and the other three groups (LDG, MDG, and HDG) using William's test. It was revealed that taking any dose of herbal drugs will improve the AA; however, only the frequency of prescription of about three capsules/day could significantly improve the levels of AA (*P* < 0.05). We conclude that that AA gain occurs in a dose-dependent manner; the higher the dose taken, the greater the AA value. We suggested that the components of mixed herbal drugs may enhance the elevation of AA [36, 37].

## 4. Discussion

Currently, nearly 2.1 billion people worldwide are estimated to suffer from presbyopia. In 2019, more than 30% of the total population in their mid-to-late 40s were reported to be affected to some degree. Surprisingly, in developing countries, the most prevalent ocular disease was found to be presbyopia in 92.5% of patients, compared with the 34% of patients reported in developed countries. With the increasing longevity of the population, most of the global population is expected to spend roughly half their lives with presbyopia [38].

Presbyopia is loss of the ability of the eye to focus sharply on nearby objects, resulting from the age-related loss of AA. The ability of the eye to accommodate or adjust its focus diminishes with age. Most of the facilities to accommodate will be lost by 55 years of age. In experiment 1, we demonstrated that the mean AA was 1.8 D in subjects aged 55 years. In 60-year-old subjects, the mean AA was 1.0 D, which dropped to 0.2 D (nearly zero) in those aged 70 years. Our examination of the Taiwanese population showed similar results to those of many other studies. Likewise, Boccardo stated that AA declined from 40 to 83 years of age in the Spanish population [39]. When the eye is at rest and focused on the distance, the ciliary muscle is relaxed. If a person wants to view objects at a close distance, the ciliary muscle contracts and induces a forward movement of the lens. This phenomenon causes the bulk of the anterior ciliary body to move forward and toward the axial length, further increasing the vitreous pressure, resulting in a release in tension in the zonular fibers around the lens equator [7]. The elastic lens capsule is able to mold the young and soft lens into a more spherical form (increasing the lens thickness) for AA gain. However, these mechanisms are subject to a series of pathologic changes during aging. We could observe the hypertrophy of the ciliary muscle, loss of the elasticity of zonular fibers and lens capsule, insertion of the ciliary muscles becoming more tangential to the lens surface, shallow A/C, IOP elevation, and hardening of the lens. Presbyopia may also occur due to the crowing of the posterior chamber and a reduction in the tension of zonules fibers at the lens equator. Therefore, the primary cause of the onset of presbyopia is the subject of aging. At times, it has also been connected to long-term reading or working at near distances, geographic latitude, higher environmental temperature, race, gender, excessive ultraviolet radiation, chronic deficiency of essential amino acids, and exposure to hair dye [39–41]. In addition, people with uncorrected hyperopia and anisometropia and contact lens wearers have shorter times until the onset of presbyopia [42]. It appears that nutrient intake—for example, vitamin C supplementation—may delay the occurrence of early age-related lens opacity and loss of accommodation. Early-onset presbyopia was recently studied in special groups, including computer workers and smartphone overusers, who were around 35 years old. Moreover, we demonstrated that the relationship between ocular floater and posterior vitreous detachment (PVD) is close. PVD may lead to symptomatic vitreous opacity, which is also related to the onset of presbyopia because of vitreous liquefaction. The peak incidence of PVD occurs between 45 and 65 years, which is when a higher prevalence of presbyopia also occurs [43].

Presbyopia is believed to occur due to a loss of visual accommodation due to the weakening of the ciliary muscle-lens zonules apparatus and the stiffening of the lens with age. In other words, an abnormal change in the configuration of the ciliary body leads to a change in lens thickness and elasticity of the lens capsule and crystalline lens; these associated ocular pathologies result in the development of presbyopia. In clinics, we could measure the variation in the AA for the evaluation of presbyopia. In fact, dynamic AA varies in different people, ages, ocular diseases, and treatments. If human AA is not suitable for users performing near-distance tasks, presbyopia develops, and extra AA from reading glasses or other methods is necessitated.

In experiment 1, the decline in accommodation in the subjects aged between 45 and 50 years was not obvious. The add diopters between the two groups were almost +0.70 D and +1.2 D (Table 1). In clinics, when the add power is around +1.0 D, the use of extra-additional reading glasses is not indicated. Our results were similar to the report of Pescosolido's research team [13]. In experiment 2, in the ninth month, we unexpectedly found that the mean AA still showed significant results for people aged between 65 and 70 years, who had gone without treatment for three months (*P* < 0.05) (Table 2). Why did only older people retain the apparent AA and why did most of the people from the groups aged from 65 to 70 years have improved near vision? We hypothesize that the chemical substances of herbal drugs may be metabolized in younger people rapidly; however, the pharmacologic functions remained effective in much older people. In the meantime, their near vision also remained at J 2.5 (baseline: J 5.5), and the pupil size was maintained at 4.5 mm (baseline: 4.3 mm), which, on comparison, showed significant differences. The previously mentioned findings suggest that the parasympathetic functions that were beneficial for accommodation were still functional at this point. Hence, we suggest that the oral herbal drugs from our designed formula could offer a valid improvement in AA, which would be beneficial for people with presbyopia. In experiment 3, we found an elevation in the maximal AA in the three groups with various treatments. Although the only elevated AA value in the HDG in the sixth month was remarkable (*P* < 0.05), the maximal AA in LDG and MDG also showed an elevation. This meant that taking any capsules would be good for obtaining an improved AA. According to the outcomes mentioned in Table 3, the ideal prescription indicates that the subjects should take three capsules each day for at least six months.

There are many methods for the treatment of presbyopia in patients. The strategies for dealing with presbyopia include using a separate optic device before the visual system (reading spectacle), change in the gaze to view through optical zones of different optical powers (bifocal, trifocal, or progressive spectacles), monovision (contact lenses, IOLs, and laser refractive surgery), simultaneous images (corneal inlays), pinhole depth focus expansion (IOLs, corneal inlays, and pharmaceuticals), crystalline lens softening (lasers or pharmaceuticals), and restored dynamics (accommodating IOLs, and scleral expansion) [44–46]. After various management efforts, presbyopic people feel comfortable working at near distances. The use of reading glasses is the most common and acceptable method for the correction of presbyopia. Traditional single-vision, bifocal, and progressive eyeglasses are acceptable for humans and are relatively simple [47]. Nevertheless, the associated problems included inconvenience in performing daily or athletic activities. Optically similar, simultaneous‐image contact lenses are another choice. However, age-dependent ocular changes such as the decreased muscle tonus of both the upper and lower eyelids reduced palpebral aperture, and diminished lacrimal production and tear stability may influence one's experience of wearing contact lenses. Besides this, the relatively higher infectious rate decreased stereopsis, and contrast sensitivity may limit the desire of patients to use this method [48]. Considering invasive techniques, cataract surgeries combined with multifocal IOL appear to offer the most consistent and reliable choice for presbyopia [49]. Other approaches also show promise, but as yet no method has demonstrated reliable and long-term effectiveness. Besides this, some unexpected adverse reactions and possible perpetual damage may irreversibly impact the intention of people to use these methods [44].

Pharmacologic methods are another choice for presbyopia. Most eye drops given to presbyopic patients are mixed and include compounding agents. We searched for the treatment of presbyopia in the PubMed system. To our surprise, only a few articles on topical eye drops have been published in journals until 2021. Eye drops for presbyopic subjects have become popular due to several advantages, such as their ease of use, effectiveness, and noninvasiveness, and they also do not limit patients' daily activities [50]. The pharmacological mechanisms of the drugs are to enhance the contraction of the ciliary muscle, pupil control, and management of DoF. Some eye drops may gain AA for the compensation of presbyopic loss, which should improve near vision. For example, Benoozzi and coworkers used a combination of pilocarpine (1%) and diclofenac (0.1%) (NSAID) for presbyopia. The cholinergic drug, pilocarpine, would act on the muscarinic receptors of the ciliary muscle and iris and then restore near visual acuity. In addition, the pharmaceutical form used was devoid of any inflammatory or other collateral effects through the inhibition of prostaglandin synthesis by NSAIDs [38]. The eye drops comprise several components, including pilocarpine, dapiprazole, and naphazoline, which are used alone or in combination for presbyopia. Pilocarpine can be used topically for stimulating accommodation for presbyopic subjects. Nevertheless, various concentrations have different results. One-percent pilocarpine could facilitate pupil constriction and ciliary body contraction, thus stimulating accommodation and improving tear production by stimulating lacrimal gland secretion. On using the eye drops containing the combination of 4% pilocarpine and 10% phenylephrine, the subjects' AA may even reach the maximal 14 D. It was verified that pilocarpine 6% should be at a higher concentration relative to a clinical therapeutic dose. Pilocarpine could also induce an increase in the lens thickness [51, 52]. Unfortunately, the use of pilocarpine has many side effects, such as headache, dizziness, nausea, flushing, acute-angle closure glaucoma, and retinal detachment [53]. Dapiprazole is an adrenergic *α*1 blocker that counteracts the effects of 1% tropicamide and 2.5% phenylephrine. It should reverse the mydriatic function. Hence, the presumed effects could diminish the haloes after excimer keratectomy. Moreover, dapiprazole could also increase the level of AA, DoF, and comfortable reading ability in patients with presbyopia [54]. However, we must pay attention to its side effects, such as photophobia, corneal endothelial toxicity, and poor near vision. Finally, naphazoline is a direct-acting sympathomimetic amine with vasoconstrictive activity. Upon ocular administration, naphazoline exerts a rapid effect by acting on alpha-adrenergic receptors at the conjunctiva to produce the vasoconstriction of ocular arterioles, resulting in decreased conjunctival congestion, itching, irritation, and redness. For presbyopic subjects, naphazoline intensifies the relaxing effect of pilocarpine on the dilator pupillae, increasing the Ach level and reducing norepinephrine release. Hence, it is also one type of eye drop recommended for presbyopia [55].

We also reviewed the publications on herbal treatments for presbyopia on PubMed and found only two papers. Biswas et al. conducted one herbal drop preparation to be applied by patients with various ocular diseases for six months. They found that cataracts and presbyopia became better [56]. However, the contents of the herbal drug were not mentioned, and the detailed usage was also not clear. Likewise, Khan et al. demonstrated that the “Ocucure” tablet (500 mg), including *Foeniculumvulgare* (150 mg), *Paeonia officinalis* (150 mg), *Coriandrum sativum* (100 mg), and *Benincasa hispida* (100 mg), and two tablets were prescribed daily for six to eight weeks. Presbyopic symptoms in only 17 cases (28.8%) improved when compared with the control group (six patients; 11.5%). Nevertheless, the sample size in their research was relatively small and the subjects were too young (mean age was about 33.5 years) [57]. In this research, we tried to design a new mixed herbal drug to improve AA for good near vision. During our experiments, we made mixed herbal capsules, and the pharmacological effects of each traditional Chinese drug were analyzed.

Cassiae Semen is a well-known traditional medicine that has been used for improving eyesight, liver function, and various types of inflammation in China since ancient times. Of late, cassia seeds have been used to treat headaches, obesity, periocular pain, constipation, hypertension, hyperlipidemia, Alzheimer's disease, ischemic stroke, and bronchospasm, as well as some ocular diseases such as dry eye and retinitis pigmentosa [58]. A total of 55 chemical compounds in Cassiae Semen were identified, including flavonoids, emodin, chrysophanol, physcion, obtusin, rhein, aurantio-obtusin, chryso-obtusin, and anthraquinones—which showed various pharmacological functions, including anticoagulant, antiangiogenic, antimicrobial, and antioxidant abilities. For example, physcion belongs to polyphenol, which has antioxidative and anti-inflammatory properties. Aloe-emodin regulates the apoptosis of retinal ganglion cells and prevents glaucoma. Besides this, obtusin and aurontio-obtusin may enhance vasodilation and diuresis. Chrysophanol and physcion were suggested to decrease IOP in our study in 2013 [19]. We know that shallow A/C and the elevation of IOP may be found during accommodation, resulting in periocular pain and even headaches. Therefore, the ability of cassia seeds to lower the IOP function could relieve the symptoms of presbyopia. Furthermore, obtusifolin and emodin are important in accommodation due to acetylcholinesterase (AChE) activity in the cholinergic nervous system through the activation of the muscarinic receptors [59]. We believed that the contractility of the ciliary muscle, relaxation of zonules, more curved lenses, mydriasis, increase in the thickness of the lens, and modification of the shape and position of the lens during accommodation due to parasympathetic functions may be due to the effects of cassia seed extracts. Hence, taking Cassiae Semen may help to obtain AA and good near vision. The real mechanisms behind accommodative pathways need to be investigated more exhaustively in the future.

The fruit of *L. barbarum* or goji berries has been used as an antiaging herb to maintain good health for a long time. Goji can also improve “Kidney Yang Deficiency Syndrome” and balance the “yin” and “yang” in the body. There are various primary extracts of goji berries, including carotenoids, phenolic acid, flavonoids, betaine, taurine, *β*-sitosterol, polysaccharides, scopoletin, and vitamins [60]. Goji berries exhibit cytoprotective, immunomodulatory, antifatigue, neuroprotective, anti-inflammatory, antiradiation, antiapoptotic, anticoagulant, antiplatelet, cardioprotective, antiproliferative, antimicrobial, and antioxidant effects. These berries can also improve arterial compliance, skeletal muscle power, renal function, and hemopoiesis and ameliorate anemia, asthma, metabolic syndrome, diabetes mellitus, various types of cancers, and Parkinson's disease [61]. A recent study showed that the use of goji berries led to a change in serum metabolic profiles, including energy metabolism (lactic acid), lipid metabolism (cholesterol), and biosynthesis of catecholamine (norepinephrine). Furthermore, Guo et al. demonstrated that *L. barbarum* polysaccharides could increase the level of cortisol and epinephrine [62]. In the ophthalmic field, wolfberries are known to be beneficial for presbyopia-induced dry eye, blurred vision, ocular fatigue, age-related macular degeneration, diabetic retinopathy, UV light-induced retinal degeneration, retinitis pigmentosa, and even glaucoma [63].

Dendrobium, known as “Shihu,” is a Chinese traditional medicinal herb that belongs to the *Orchidaceae* family. The stem has been traditionally used for centuries for treating diseases such as throat inflammation and chronic superficial gastritis, strengthening the body, and prolonging life. Dendrobium is widely famous since ancient times for its medical value in treating cataracts. The ingredients extracted from DD are alkaloids, stilbenoids, anthracene, polysaccharides, fluorine, flavone, phenanthrene, and gigantol that have several pharmacological functions, including enhancing immune activities, controlling blood sugar, inhibiting tumor growth, and protecting the liver from oxidative stress [64]. DD is also used for several ophthalmic diseases, such as dry eye and diabetic and ischemic retinopathy. Accumulating evidence indicates that all improvements in ocular conditions are due to the plant's anti-inflammatory and antioxidant abilities. DD also shows significant hypoglycemic and anticataract activities through its inhibition of nitric oxide (NO), aldose reductase, protein glycation, and advanced glycation end products (AGEs) [65]. During aging, the cortex thickens, the lens becomes more curved, and the nucleus becomes less deformed. The pharmacologic mechanisms of DD include the prevention of the development of cataracts and improvement in AA. Except for DD, cassia seeds and wolfberries also contribute to slowing down the formation of cataracts through their antioxidants. For example, cassia seeds contain polysaccharides, emodin, and flavonoids that may resist oxidase stress and decrease the maturation of cataracts. The anthraquinones have inhibitory activity in protein glycation and aldose reductase, preventing the formation of cataracts [66]. As for *L. barbarum*, it can induce the activation of suituin 1 and thereby decrease cataract formation [67]. The extracts from goji berries, including polysaccharides, phenolic acid, and flavonoids, can help in preventing free radicals from attacking the lens fibers. Therefore, goji berry and cassia seeds have a stronger antioxidant ability, which may retain lens clarity and prevent presbyopia.

In this study, we administered mixed herbal drugs to subjects aged 45 to 70 years for six months. The mean AA reached a maximum value of 2.1 D after the sixth month (*P* < 0.05), and this decreased to 2.0 D at the ninth month without herbal treatments (*P* < 0.05). This indicates that these drugs could still exert positive effects after taking them for six months and further for at least three months without drug intake. The outstanding pharmacologic effects are prolonged because of the increased AA gain. Besides this, pupil size and near vision reached their maximal levels (5.6 mm and J 1.5) in the sixth month. The effective ability of mydriasis and near vision (4.2 mm and J 2.5) was also noted after the ninth month. We suggest that the parasympathetic function is predominant even in the ninth month. The primary pharmacological effects of the improvement in near vision were due to accommodation rather than miosis. A smaller pupil-enhanced DoF (sympathetic function) and good accommodative ability may increase the AA more (parasympathetic function). Cassiae Semen could enhance the accommodation by the parasympathetic effects that obtain AA. Besides this, intake of cassia seeds leads to miosis, followed by the pinhole effect and DoF, and goji berries supply sympathetic effects for dilated pupils. It is interesting that cassia seeds have parasympathetic effects; however, *L. barbarum* in our herbal capsule showed sympathetic effects. We conducted several previous studies and found that the ideal ratio of cassia seeds and *L. barbarum* was 5 : 5, to affect the presbyopic participants to have the best near visual acuity, with dilated pupils. In other words, the parasympathetic function is predominant when taking the designed herbal capsules, while the ratio of cassia seeds and *L. barbarum* was 5 : 5 in our previous small sample of studies after many adjustments (not published yet). The partial ability of the sympathetic function of goji berries may be counteracted by the parasympathetic function from Cassiae Semen. However, just the combined concentrations of two herbal drugs could enhance the near vision at around J 1.5. DD has an anticataract ability. In this study, we used three herbal drugs to offset the decrease in AA in presbyopic subjects. According to past studies, the best-estimated ratio of cassia seeds and *L. barbarum* was 5 : 5. When we added DD, we found the golden ratio of cassia seeds, goji berries, and DD to be 5 : 5:1. The final weight was 200 mg of cassia seeds, 200 mg of goji berries, and 40 mg of DD in one capsule, which may aid in improved AA of the participants.

In general, the symptoms of presbyopic patients worsen significantly between 40 and 50 years of age, with patients complaining about blurred vision at their usual reading distance, ocular strain, and periocular pain. Sometimes, dry eye, diplopia, chromatic aberrations, and even headaches may worsen under dim environments (named night presbyopia). At present, 90% of smartphone users are prone to digital eye strain (so-called computer vision syndrome; CVS) and presbyopia in modern society. Recently, Iqbal et al. reported that smartphone misuse is the main cause of the development and prevalence of CVS among users. Furthermore, through their documented multifocal electroretinogram (mfERG) examinations, they also proved that CVS elicits screen-induced foveal dysfunction. These visual sequelae might also cause simulated near vision troubles related to presbyopia [72]. The occurrence of digital eye strain is similar to presbyopic problems. When viewing examinations, or something, especially for a long period of time, the presbyopic symptoms easily developed and were accompanied by the weakness of the orbicularis oculi muscle and decreased lacrimal gland secretion, which eventually leads to a reduction in blinking reflex and in dry eyes [68–70]. The highest prevalence of presbyopia is in middle-aged patients with decreasing lacrimal function. These individuals always read or work at near distances for long periods of time, which leads to a diminished blinking rate and worsened dry eyes. The pathological mechanisms include the instability of the tear film, tear hyperosmolarity, oxidative stress, and inflammation of the ocular surface [71]. Hence, the administration of both artificial tears and anti-inflammatory drug is prescribed for dry eyes. We propose that polysaccharides in goji berries can enhance the anti-inflammatory functions, inhibiting free radicals and reducing oxidase stress, while betaine protects the cornea from environmental stress and improves the moisture and nutritional levels in eyes experiencing presbyopia and associated symptoms in our research [20].

Our study also had a few limitations. First, because the number of participants was so large (400), we adopted the subjective method such as push-up test for measuring AA, which was simple and rapid during the experiments. Indeed, various subjective techniques are inadequate for the evaluation of the AA; objective ways such as dynamic retinoscopy may aid in achieving more exact AA. However, it wastes time and involves high technology of performance, which is not suitable for the large sample study. If possible, we could take the subjective and objective AAs simultaneously and average to obtain more exact data. Second, we categorized the participants according to age intervals of five years (between 45 and 70 years), although the changes in presbyopia sometimes varied. Clinically, for some subjects with long-term near work, their AA changes within two to three years rather than five years, and we could not take that into consideration and could only adopt the five-year interval for rough average.

Finally, in our series of experiments, we formulated a mixed herbal capsule that seems to benefit the younger groups that use smartphones and older groups with long-term work and loss of the accommodation ability. We have the desire to manufacture the capsules to help the subjects with difficulty in near working in the future.

## 5. Conclusion

Presbyopia describes the progressive loss of accommodation, weakness of the contraction of the ciliary muscles, reduction in the elasticity of zonules and lens capsules, and increased stiffness of the lens. The mechanism of our designed treatment is based on enhancing the accommodative ability and pupil control. Besides this, ameliorating the developing cataract formation is also important.

In this study, we proposed a novel herbal combination including Cassiae Semen, wolfberry, and DD for use in presbyopia. The parasympathetic function of cassia seeds could enhance the accommodative system, and the goji berry could supply appropriate effects for the sympathetic nervous system and moisturizing effects for the presbyopic symptoms and associated dry eyes. DD diminishes the progression of cataracts through the inhibition of sorbitol and AGE accumulation, and Cassiae Semen and wolfberry exhibit strong antioxidant abilities. The success rate was approximately 95% after six months of therapy. Therefore, we suggest that herbal drug supplements may be another choice because of their convenience, safety, and persistence of their beneficial pharmacologic functions.

## Figures and Tables

**Table 1:** Demographics of the volunteers in experiment 1.

Age	Baseline		
	Numbers	AA	Presbyopic D
45	40	3.6 ± 0.2D	+0.7 ± 0.1D
50	200	2.6 ± 0.1D	+1.2 ± 0.1D
55	40	1.8 ± 0.2D	+1.6 ± 0.1D
60	40	1.0 ± 0.1D	+2.0 ± 0.1D
65	40	0.3 ± 0.1D	+2.4 ± 0.2D
70	40	0.2 ± 0.1D	+2.3 ± 0.1D

(1) Numbers: a total of 400 participants were gathered through an interview in our study aged between 45 and 70 years. Besides this, 240 subjects were enrolled in experiment 2 and 160 participants were separated for experiment 3 for persons with a mean age of 50 years. (2) At first, we gained the AA from subjects with different ages using the subjective push-up method. Subsequently, AA may be converted to the additional diopters of presbyopic reading glasses. (3) Near vision: test at 40 cm in front of the volunteers' eyes. (4) Age (mean age) in years. (5) AA: amplitude of accommodation. (6) D: Diopter.

**Table 2:** The changes in AA of various ages after talking herbal drugs in experiment 2.

Age	Times				
	Baseline	3^rd^ month	6^th^ month	9^th^ month	12^th^ month
45	3.6 ± 0.2D	3.8 ± 1.4D	4.2 ± 1.5D^*∗*^	4.0 ± 1.9D	3.7 ± 1.5D^#^
50	2.6 ± 0.1D	2.9 ± 0.5D	3.1 ± 0.9D^*∗*^	2.9 ± 0.9D	2.7 ± 2.0D^#^
55	1.8 ± 0.2D	2.0 ± 0.8D	2.3 ± 1.5D^*∗*^	2.3 ± 1.1D	1.9 ± 1.2D^#^
60	1.0 ± 0.1D	1.2 ± 0.6D	1.6 ± 0.9D^*∗*^	1.3 ± 0.6D	1.0 ± 0.6D^#^
65	0.3 ± 0.1D	0.5 ± 0.2D	1.0 ± 0.5D^*∗*^	0.9 ± 0.6D^*∗*^	0.4 ± 0.2D^#^
70	0.2 ± 0.1D	0.3 ± 0.2D	0.6 ± 0.3D^*∗*^	0.5 ± 0.2D^*∗*^	0.2 ± 0.2D^#^

(1) All 240 participants were recruited in experiment 2, and a total of 40 volunteers with various ages took part in each group between 45 and 70 years. (2) All the volunteers took the capsules three times daily for six months and stopped treatment at the end of the sixth month. Afterward, we followed up the parameters and questions for a further six months. (3) A herbal capsule included 200 mg Cassiae Semen, 200 mg wolfberry, and 40 mg *Dendrobium huoshanense* (DD). (4) At first, we compared the results (at the third, sixth, ninth, and twelfth months) with the baseline. Second, we inspected the data (at the ninth and twelfth months) with the results of the sixth month. In all comparisons, the method of Scheffe's test was used for analysis. (5) When the *p* value was less than 0.05, it was considered as a significant difference. Hence, in the first comparison, it will be marked as ^*∗*^, and in the second comparison, it will be marked as^*#*^.

**Table 3:** The outcomes after taking various amounts of herbal drugs in experiment 3.

Age	Times				
	Baseline	3^rd^ month	6^th^ month	9^th^ month	12^th^ month
Placebo	2.6 ± 0.1D	2.6 ± 0.4D	2.6 ± 0.5D	2.6 ± 0.5D	2.5 ± 0.6D
1 cap/day	2.6 ± 0.1D	2.7 ± 0.8D	2.8 ± 0.4D	2.8 ± 0.9D	2.6 ± 0.4D
2 cap/day	2.6 ± 0.1D	2.8 ± 1.1D	2.9 ± 0.8D	2.9 ± 0.6D	2.7 ± 0.8D
3 cap/day	2.6 ± 0.1D	2.9 ± 0.9D	3.2 ± 0.5^%^^*∗*^	3.0 ± 0.5D^*∗*^	2.7 ± 0.7D^#^

(1) In experiment 3, 120 volunteers with a mean age of around 50 years were involved, and they were randomly divided into four groups. (2) Group 1 (placebo group) (*n* = 20): only 10 mg vitamin C daily was taken. Group 2 (*n* = 20): all subjects took one capsule/day. Group 3 (*n* = 20): all victims took two capsules daily. Group 4 (*n* = 20): total volunteers were fed with three capsules in each day. (3) All volunteers took the various doses of mixed herbal capsules for six months in groups 2, 3, and 4 and stopped treatment at end of the sixth month. Then, the participants received follow-up for the next six months. The associated parameters and questions were collected. (4) At first, we compared the AA of at the third, sixth, ninth, and twelfth months with the baseline using Scheffe's test. Moreover, the results at the ninth and twelfth months were recorded and compared with the outcome of the sixth month by Scheffe's test. Third, the mean AA in group 2 (1 cap/day), group 3 (2 cap/day), and group 4 (3 cap/day) was compared with the placebo group using William's test. (5) A *P* value less than 0.05 was considered significantly different. Hence, in the first comparison, it will be marked as^*∗*^, in the second comparison, it will be marked as^*#*^, and in the third comparison, it will be marked as ^%^. (6) Cap/day: capsule(s)/day.

## Data Availability

The datasets used during the current study are available from the corresponding author.
